# Congenital Gastrointestinal Malformations in a Romanian Tertiary Centre (2020–2024): A Retrospective Cohort Study of Diagnosis, Distribution, and Outcomes

**DOI:** 10.3390/diagnostics16091408

**Published:** 2026-05-06

**Authors:** Iulia Stratulat-Chiriac, Raluca Ozana Chistol, Lăcrămioara Perianu, Elena Țarcă, Viorel Țarcă, Alina Mariela Murgu, Paula Popovici, Ioana-Alina Halip, Elena Cojocaru, Valeriu Chisălău, Cristina Furnică

**Affiliations:** 1 Grigore T. Popa University of Medicine and Pharmacy, 700115 Iasi, Romania; chiriac.iulia@d.umfiasi.ro (I.S.-C.); raluca-ozana.chistol@umfiasi.ro (R.O.C.); lacrima12@gmail.com (L.P.); alina.murgu@umfiasi.ro (A.M.M.); paula.popovici@umfiasi.ro (P.P.); alina-ioana.grajdeanu@umfiasi.ro (I.-A.H.); elena2.cojocaru@umfiasi.ro (E.C.); cristina.furnica@umfiasi.ro (C.F.); 2 Faculty of Medicine, Apollonia University, Strada Păcurari nr. 11, 700511 Iasi, Romania; viorel.tarca@univapollonia.ro; 3 “Piatra Neamț” County Emergency Hospital, 610136 Piatra Neamț, Romania; valeriuchisalau@gmail.com

**Keywords:** congenital gastrointestinal malformations, congenital digestive tract anomaly, prenatal diagnosis, neonatal surgery, Romania, mortality

## Abstract

**Background/Objectives:** Congenital gastrointestinal malformations (CGIMs) are important causes of neonatal surgical morbidity with potential long-term consequences. Although some can be suspected on prenatal ultrasound, data on their clinical spectrum, burden, and distribution remain limited in Eastern Europe. This study aimed to describe the diagnostic spectrum, timing of diagnosis, documented prenatal ultrasound suspicion, and the early outcomes of CGIMs managed at a Romanian tertiary referral centre between 2020 and 2024, a period overlapping the COVID-19 pandemic. **Methods:** We conducted a retrospective, single-centre observational study including all consecutive paediatric patients with a CGIM admitted between January 2020 and December 2024. Cases were analysed by index anatomical diagnosis, phenotypic complexity, and etiologic background. Logistic regression was used to examine factors associated with documented prenatal suspicion and in-hospital mortality, and annual hospital-based CGIM admission rates were modelled with Poisson regression, using the number of paediatric surgical admission as the offset. **Results:** Among the 231 children (58.9% male), the most frequent diagnoses were persistent omphalomesenteric duct remnants (16%), oesophageal atresia with or without tracheoesophageal fistula (15.6%), and anorectal malformations (13.9%). Documented prenatal ultrasound suspicion was present in 17.7% of pregnancies (41/231) and was likely underestimated because antenatal documentation was unavailable for 17.7% of the cohort. The highest proportions of documented prenatal suspicion were observed in jejuno-ileal and duodenal atresia. Foregut malformations were the most common by embryological grouping (93/231, 40.3%). Most cases were diagnosed during the neonatal period (n = 161, 69.7%). CGIM admission rates per 1000 surgical admissions ranged from 20.8 to 38.2. An exploratory Poisson model yielded a hospital-based rate ratio per calendar year of 0.88 (95% CI 0.81–0.97; *p* = 0.008). However, given the limited number of annual observations and increasing total surgical admissions, this finding should be considered exploratory and hypothesis-generating only. Complex cases accounted for 8.2% and associated extra-intestinal anomalies were present in 70.1%. In-hospital mortality was 13.0% and occurred predominantly in patients with complex or foregut malformations. In the primary complete-case multivariable model, prematurity remained independently associated with mortality, whereas complex CGIMs were not independently associated with mortality after adjustment. A prespecified multiple-imputation sensitivity analysis yielded a stronger estimate for complex CGIMs, but this finding was interpreted cautiously and not treated as a primary result. **Conclusions:** In this tertiary referral cohort, documented prenatal suspicion of CGIM was low and strongly diagnosis-dependent, while most cases were identified in the neonatal period. Mortality was concentrated in foregut and clinically complex presentations in the descriptive analysis, while prematurity remained independently associated with death in the primary multivariable model. These findings highlight the need to strengthen prenatal referral pathways and coordinated national surveillance.

## 1. Introduction

Congenital gastrointestinal malformations (CGIMs) are a heterogeneous group of developmental disorders affecting the structure and function of the digestive tract. They vary widely in anatomical location, clinical severity, timing of presentation, and outcomes. Taken together, they represent an important component of neonatal and paediatric surgical pathology, but their incidence varies widely depending on the specific anomaly. Reported incidence varies across conditions, from relatively common anomalies such as hypertrophic pyloric stenosis to rare entities such as cloacal malformation [1]. The most common anomalies diagnosed in the neonatal period are anorectal malformations (2–6 per 10,000 live births), followed by oesophageal atresia with or without tracheoesophageal fistula (2–4 per 10,000 live births), and Hirschsprung disease (1–2 per 10,000 live births) [2,3]. Cloacal exstrophy remains one of the rarest entities (1 per 200,000–400,000 live births) [4].

The clinical burden associated with CGIM is equally variable. The disease spectrum ranges from isolated defects with favourable outcomes after surgical correction to complex malformations associated with adverse neonatal surgical outcomes, prolonged multidisciplinary care, and long-term morbidity [5,6].

Despite advances in foetal screening programs and antenatal imaging, prenatal detection of CGIM remains variable and strongly dependent on anomaly type, particularly in areas with heterogeneous access to specialist imaging and follow-up [7,8]. Across Europe, registry-based studies have reported marked heterogeneity in the prevalence, distribution, and timing of diagnosis of CGIMs, both antenatal and postnatal, influenced by differences in economic settings, healthcare infrastructure, and antenatal screening practices [9,10]. Comparisons across countries are further influenced by variation in pregnancy termination legislation, coding and reporting practices, and discrepancies between major classification systems—such as ICD–10 and EUROCAT [11,12].

Given this heterogeneity, analysing CGIMs solely by diagnosis may be insufficient. To address this, we examined case presentation through three complementary domains: index anatomical diagnosis, phenotypic complexity, and documented etiologic background. This framework was used to interpret clinical presentation as the result of interacting developmental, anatomical, and contextual factors, and to relate these domains to prenatal suspicion, timing of diagnosis, associated anomalies, and early outcomes. Beyond the ten index diagnostic groups, cases were classified as isolated gastrointestinal malformations or complex cases. For inferential statistics, complex cases were retained as a predefined category to reflect increased anatomical and surgical challenges. Etiologic categories were reviewed descriptively and included chromosomal, monogenic, association/sequence-related, and multifactorial or unknown conditions when no specific diagnosis was documented. 

The study period (2020–2024) spans a phase of healthcare disruption and subsequent normalization [13,14]. We examined whether prenatal detection and case volume changed over time. Evidence from Eastern Europe remains limited, hindering regional benchmarking and policy development [15,16]. To address these gaps, we conducted a five-year retrospective study at a Romanian tertiary referral centre to examine how clinical and referral-context factors relate to prenatal suspicion, timing of postnatal diagnosis, and early outcomes in a hospital-based referral cohort.

## 2. Materials and Methods

### 2.1. Study Design and Setting

This retrospective observational study aimed to investigate hospital-based temporal trends in CGIM admissions and factors associated with diagnosis and outcomes. This study was conducted at the Emergency Hospital for Children “Saint Mary” in Iasi, Romania, the main tertiary paediatric referral centre for northeast and southeast regions. We included all consecutive paediatric patients aged 0–18 years admitted with congenital gastrointestinal malformations (CGIMs) between 1 January 2020 and 31 December 2024. 

The Emergency Hospital for Children “Saint Mary“ is a paediatric-only institution and does not provide obstetric services; therefore, all neonatal patients in this cohort were delivered elsewhere (i.e., all cases were outborn); referrals occurred postnatally after birth facility assessment and stabilisation. The centre receives referrals from a multi-tier maternity network comprising approximately 23–25 obstetric units (depending on annual reporting variability), across the northeast and southeast regions of Romania, including primary-, secondary-, and tertiary-level facilities. Only two public maternities, located in the same city as the surgical tertiary centre, provide tertiary-level neonatal intensive care, including specialised preterm care, and serve as key referral centres for high-risk pregnancies. 

The regional maternity system is predominantly hospital-based, with the vast majority of deliveries occurring within obstetric units. Home births represent a negligible proportion of deliveries; in our cohort, births occurring outside a hospital were uncommon. According to data from the National Institute of Statistics of Romania, the counties included in this study reported roughly 43,000–48,000 live births annually between 2020 and 2024, resulting in an estimated total of 226,600 births over the study period. These figures were included only to contextualise the approximate catchment population covered by the referral network and were not included in the analysis.

Antenatal imaging is delivered through a mixed public–private system without a centralised registry. The number of ultrasound providers cannot be reliably quantified at the regional level. In addition, access to detailed prenatal ultrasound documentation and the transfer of data at the referrals are inconsistent across facilities, which may influence the recording of antenatal suspicion in tertiary paediatric admission records. 

Certain congenital gastrointestinal anomalies, particularly foregut and midgut atresias, are frequently associated with polyhydramnios and an increased risk of preterm delivery. These antenatal features may prompt suspicion and referral to tertiary maternity units within the region for further evaluation and perinatal management. Referral of neonates to the tertiary surgical centre occurs postnatally after the clinical diagnosis and initial stabilisation at the birth facility. Antenatal suspicion may lead to delivery planning in a tertiary maternity unit, whereas postnatal referral to the paediatric surgical centre typically occurs after birth. These clinically relevant characteristics were partially captured in the dataset through documentation of polyhydramnios and gestational age at birth (including prematurity status), where available. In contrast, detection of more distal gastrointestinal anomalies is variable and often limited, contributing to differences in referral timing and documentation. Because external ultrasound reports were inconsistently transferred, some pregnancies with true prenatal suspicion may have been classified as having no documented antenatal suspicion. This likely introduced non-differential misclassification of prenatal suspicion, which would be expected to attenuate associations and underestimate the documented rate of prenatal suspicion. Consequently, our measures of prenatal suspicion should be interpreted as conservative. 

**Figure 1:**
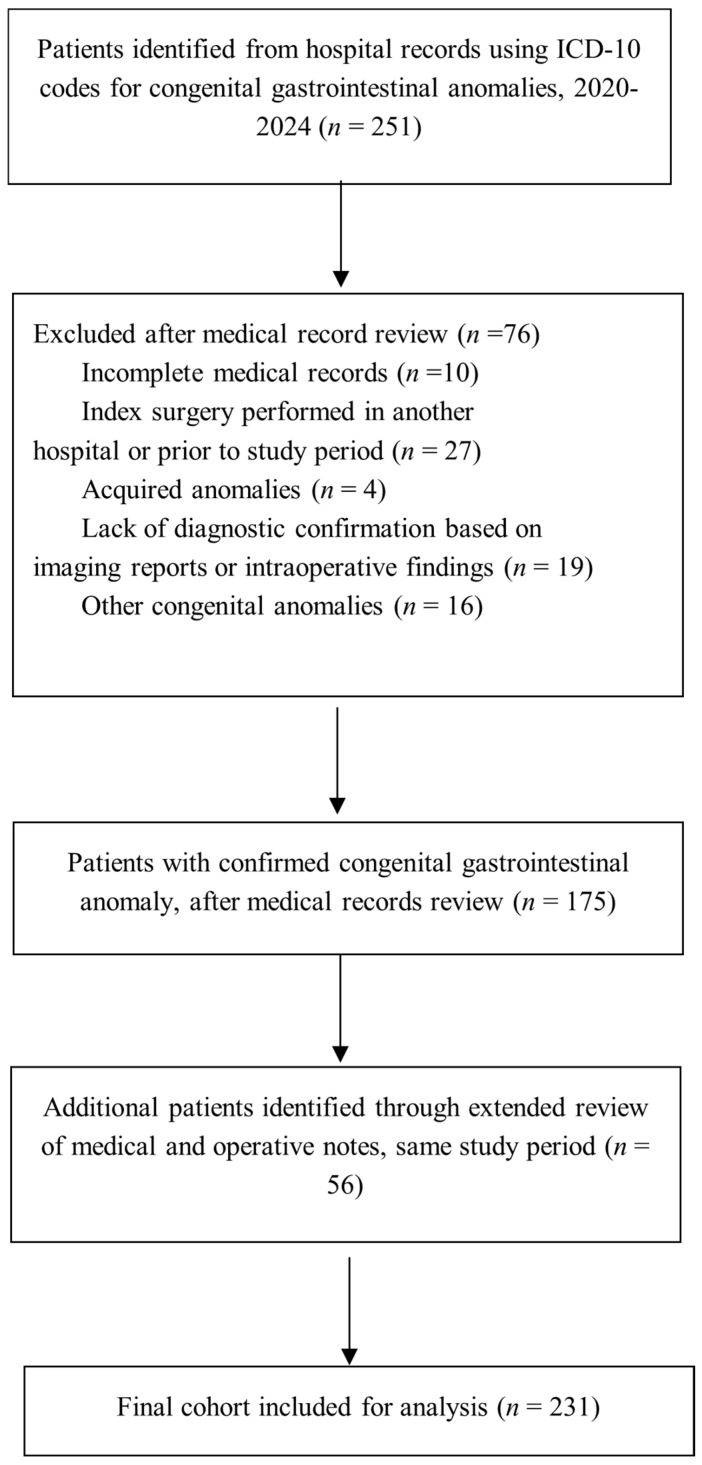
Flow diagram of patient selection and diagnostic confirmation in a retrospective cohort study.

Following ethics approval, we identified eligible cases by screening the institutional electronic medical records using the International Classification of Diseases (ICD)–10 codes (chapter XVII, congenital malformations) for congenital gastrointestinal anomalies (Supplementary Data) [17]. This search yielded 251 cases, and their clinical and operative notes were manually reviewed to confirm eligibility. Of these, 175 patients met the inclusion criteria, and 76 were excluded. To reduce under-ascertainment related to administrative coding inaccuracies, we expanded manual screening of medical and surgical notes for the same study period and identified an additional 56 eligible patients, yielding a final cohort of 231 children (Figure 1). No additional exclusions were made after eligibility was confirmed. A prospective registry was initiated in June 2024 following institutional ethics approval; however, all analyses in this report used retrospectively abstracted data. No a priori sample size calculation was performed due to the retrospective design.

All reported proportions and rates are hospital-based, using paediatric surgical admissions as a denominator; they should not be interpreted as population incidence.

This retrospective observational study was conducted and reported in accordance with the STROBE (Strengthening the Reporting of Observational Studies in Epidemiology) guidelines [18].

### 2.2. Eligibility Criteria

Eligible patients were aged 0 days to 18 years with at least one congenital gastrointestinal anomaly. Primary gastric volvulus (Q40.2) was included only when intraoperative findings confirmed congenital hyperlaxity or the absence of gastric ligaments, in the absence of acquired causes like prior surgery, adhesions, or trauma.

We excluded isolated abdominal wall defects, congenital diaphragmatic hernia with secondary malrotation, congenital anomalies of the liver, biliary tree, and pancreas, acquired gastrointestinal conditions, incomplete surgical data, or cases treated surgically in another institution (except diagnostic biopsy for Hirschsprung disease). These exclusion criteria were applied to maintain consistency in diagnostic classification and clinical pathways and reduce confounding factors related to distinct embryological origins and surgical management approaches.

The congenital gastrointestinal malformations in the final analysis were: oesophageal atresia with/without tracheoesophageal fistula (Q39.0–Q39.2), congenital hypertrophic pyloric stenosis (Q40.0), duodenal atresia/stenosis (Q41.0), malrotation with/without volvulus (Q43.3), intestinal atresia/stenosis (Q41.1-jejunal, Q41.2-ileal, Q41.8-ileo-jejunal), omphalomesenteric duct remnants (Q43.0, most commonly Meckel’s diverticulum), Hirschsprung disease (Q43.1), anorectal malformations (Q42.0–Q42.3), cloacal malformations (Q42.7), duplication cyst (Q43.4), and congenital gastric volvulus (Q40.2). In total, 231 patients met the inclusion criteria and were enrolled in this study. Collected data included demographic and perinatal characteristics (including gestational age, where available), antenatal findings (including documentation of polyhydramnios when reported on a prenatal ultrasound), diagnostic characteristics (primary diagnoses, associated congenital malformations [gastrointestinal or other organs], the embryological segment affected, clinical presentation), and outcomes (status at discharge, follow-up period, number of hospital admissions until discharge, or the last admission). Only the index admission for the index condition was included for annual case counts; readmissions for follow-up or complications were included in the descriptive analysis but were not counted as incident cases.

### 2.3. Definitions

When multiple CGIMs were present, the index anomaly was defined as the most proximal lesion anatomically if diagnosed during the same admission; if lesions were diagnosed at different dates, the index anomaly was the earliest confirmed diagnosis. Prenatal ultrasound suspicion (hereafter, prenatal suspicion) was defined as recorded prenatal ultrasound documentation available in the patient’s hospital file that was suggestive of a gastrointestinal malformation confirmed postnatally by imaging and/or intraoperative findings. Antenatal information, including ultrasound findings, was extracted from maternal or referral documents within the child’s hospital records. Ultrasound status was categorised as: (i) report available, (ii) documentation stating that no ultrasound was performed, or (iii) no antenatal documentation available in the medical reports (neither an ultrasound report nor an explicit statement regarding a prenatal ultrasound). The latter category reflects missing record-based information and cannot be used to infer antenatal care [19].

Age at index admission was defined as the age in days at the first hospital admission related to the index anomaly. Prematurity (gestational age <37 weeks) was included as a clinical variable and analysed as a covariate in outcome models. For analytical purposes, diagnoses identified were classified into ten diagnostic subgroups based on the index anomaly, even when patients had multiple hospitalisations during the study period. The type of admission was classified as elective or urgent. We grouped gastrointestinal malformations according to the embryological segment affected (foregut, midgut, hindgut derivatives), based on the standard subdivision of the primitive gut.

Cases were analysed with respect to anatomical diagnosis, phenotypic complexity, and etiologic or syndromic background. These domains were treated as complementary rather than interchangeable: index anatomical diagnosis described the main gastrointestinal congenital anomaly, phenotypic complexity reflected the clinical and surgical burden of multiple gastrointestinal anomalies, and etiologic/syndromic background referred to documented underlying diagnoses when available. These domains were used as an interpretative framework to distinguish the index anatomical anomaly from the severity of phenotypic presentation and from documented etiologic background. 

The cases were classified as “complex” if they had at least two eligible congenital gastrointestinal anomalies identified during the study period and/or one eligible congenital gastrointestinal malformation associated with an abdominal wall defect (omphalocele or gastroschisis). This composite definition was operationalised as a single binary variable and used in inferential statistics. In patients with complex CGIMs, the assigned category reflected the index lesion. 

Anomaly burden was defined as the number of associated congenital non-gastrointestinal anomalies per patient. Syndromic cases were defined as patients with a documented diagnosis of a genetic syndrome, recognised malformation sequence, or association. These included chromosomal abnormalities (e.g., trisomy 18/21), well-defined syndromes (e.g., achondroplasia, foetal alcohol syndrome), and recognised associations or sequences (e.g., VACTERL association, Pierre Robin sequence). A follow-up period was available for patients who had more than one surgical admission.

For the purposes of analysis, the principal diagnoses were classified into ten distinct categories as follows: (1) oesophageal atresia +/− tracheoesophageal fistula; (2) duodenal atresia or stenosis; (3) hypertrophic pyloric stenosis; (4) intestinal malrotation +/− volvulus; (5) jejuno-ileal atresia or stenosis; (6) persistent omphalomesenteric duct remnants; (7) Hirschsprung disease; (8) cloacal malformation; (9) anorectal malformations; and (10) other anomalies (e.g. duplication cyst, gastric volvulus).

### 2.4. Outcomes

The primary outcomes were the proportion of paediatric surgical admissions accounted for by congenital gastrointestinal malformations during the study period, prenatal suspicion (documented prenatal suspicion of a congenital gastrointestinal malformation confirmed postnatally by imaging and/or intra-operative findings), and timing of diagnosis, measured as age at index admission. Prenatal suspicion was analysed as the proportion of patients, stratified by malformation type, embryological segment, and calendar year (2020–2024), with comparisons between early (2020–2021) and later (2022–2024) study periods to explore temporal differences.

Secondary outcomes described the clinical spectrum and associated features of congenital gastrointestinal malformations. These included the diagnostic spectrum of congenital gastrointestinal anomalies, expressed as the frequency and proportion of cases by anomaly type and embryological segment affected; phenotypic presentation summarised descriptively according to isolated vs. complex cases; associated congenital anomalies by organ system; documented etiologic or syndromic background; factors associated with prenatal suspicion (syndromic status, anomaly complexity, embryological segment and maternal characteristics); and in-hospital mortality. We considered a predefined set of covariates as potential confounders or proxies for confounding; where relevant, selected variables were derived from secondary outcomes to reflect underlying clinical severity and diagnostic pathways. All proportions are hospital-based and do not represent population incidence. Because no live-birth denominator or linked population-based registry was available, incidence and true prenatal detection rates could not be calculated. 

### 2.5. Quality Control

Data extraction was performed independently by two reviewers using a standardised protocol to reduce errors and bias. Discrepancies identified between the two datasets were resolved by consensus. To ensure data integrity and verify completeness, a random audit of approximately 10% of the records was conducted. 

### 2.6. Bias

Selection bias was limited by the consecutive inclusion of all eligible cases. Information bias was minimised through standardised retrospective data abstraction using predefined variable definitions and cross-verification of key variables. Confounding was addressed using prespecified covariates and multivariable models where appropriate, although residual confounding related to referral patterns and incomplete external documentation may remain. Notably, 24% of cases were identified only through manual screening of medical notes, indicating that ICD-10 codes alone would have missed a substantial proportion of eligible cases. This likely reflects variability and misclassification in administrative coding and should be considered when interpreting hospital-based data.

Information bias is most relevant for prenatal suspicion, which depended on the transfer of antenatal ultrasound findings to the tertiary-centre records. External ultrasound reports were not consistently available, particularly for referrals without attached documentation from other facilities, likely leading to under-ascertainment and under-estimation of prenatal suspicion. Ascertainment varies widely by condition. Anomalies with characteristic obstructive sonographic signs, such as duodenal or jejuno-ileal atresia associated with polyhydramnios, are more likely to be documented and referred for further management to tertiary maternity services. In contrast, conditions rarely detected antenatally (e.g., hypertrophic pyloric stenosis, anorectal malformations) likely have both low true prenatal detection and incomplete documentation. Consequently, incomplete documentation likely resulted in non-differential misclassification, which tends to bias associations toward the null. In practice, this means that our estimate of prenatal detection is likely conservative [20].

Region-level denominators (e.g., total deliveries, the number of ultrasound providers, and facility-level imaging capacity) were not linkable within the hospital record system. As a result, the findings are based on a tertiary referral cohort and may not reflect population incidence or the overall volume of regional maternity services. 

Because gestational age was unavailable for 41/231 patients, complete case analysis was used, and this may affect the estimates. 

### 2.7. Statistical Analysis

All statistical analyses were performed using SPSS Statistics version 31 (IBM Corp., Armonk, NY, USA). Descriptive analyses were performed using all available data, with variable-specific denominators reported. Continuous variables were assessed for distributional assumptions and are presented as mean (SD), or median (IQR), as appropriate. For selected skewed variables, minimum–maximum values are also provided to show the full observed spread. Categorical variables are reported as counts and percentages (*n*, %). Group comparisons used Pearson’s chi-square (χ2) test or Fisher’s exact test for categorical variables, and the Mann–Whitney U test and the Kruskal–Wallis H test for continuous variables. Effect sizes were expressed as Cramer’s V for nominal variables and epsilon-squared (e2) for nonparametric comparisons.

In addition to anatomical analysis, descriptive analyses were organised according to phenotypic complexity and documented etiologic background in order to separate anatomical distribution from anomaly multiplicity and underlying cause. Because several etiologic subgroups were small, these categories were analysed descriptively and were not entered as primary predictors in multivariable regression models. 

Multivariable logistic regression was used to identify factors associated with prenatal suspicion and in-hospital mortality, with results reported as odds ratios (ORs) with 95% confidence intervals (CIs). For the prenatal ultrasound model, antenatal care variables were available for 190/231 (82.3%) patients; therefore, regression analyses were conducted as complete-case analyses (*n* = 190). Missing prenatal information was not treated as a standard partially observed covariate because it arose from two distinct mechanisms in the referral-based dataset: an incomplete transfer of external antenatal documentation and structural non-applicability when no prenatal ultrasound had been performed. Because these states are analytically distinct, imputing prenatal variables could have introduced misclassification by conflating unavailable documentation with the absence of prenatal suspicion. We therefore retained complete-case analysis for the primary prenatal model as the more transparent approach. To quantify the potential impact of missing antenatal documentation, we also performed deterministic scenario analysis for the 41 cases without antenatal documentation. Using the full cohort denominator (*n* = 231), we recalculated the documented prenatal suspicion proportion under three assumptions: no additional prenatally suspected cases, 20 additional suspected cases, and all 41 cases suspected prenatally. This analysis was descriptive and was not used to reclassify cases in the multivariable prenatal-suspicion model. 

Given the limited number of events and the need to preserve an adequate events-per-variable ratio, sex was not included in the primary multivariable mortality model and instead, was evaluated in a secondary model. 

Mortality was analysed using multivariable logistic regression. Prespecified predictors included prematurity (yes/no), the presence of complex gastrointestinal anomalies (yes/no), and embryological segment (foregut [reference], midgut, hindgut), with the latter entered as two indicator variables. Because gestational age was missing for 41/231 (17.7%) patients, the primary mortality model was conducted as a complete-case analysis (*n* = 190; deaths = 27). Given the limited number of events (events-per-parameter = 27/4 = 6.75), model complexity was restricted a priori and interaction terms were not evaluated [21].

To assess the robustness of mortality findings and reduce potential bias from the complete-case analysis, multiple imputation by chained equations (MICE, m = 20) was applied to missing gestational age under a missing-at-random (MAR) assumption, conditional on observed variables. This imputation analysis was prespecified as a sensitivity analysis, with the complete-case model retained as the primary analysis. The MAR assumption was considered plausible, given that missingness was primarily due to incomplete documentation rather than clinical severity; however, it cannot be formally tested using the observed data. The imputation model included all variables from the mortality regression model, as well as the mortality indicator, to preserve associations between covariates and the outcome. Imputation was restricted to gestational age (and derived prematurity variable); mortality and other outcome variables were not imputed. Logistic regression was performed within each imputed dataset, and pooled estimates were obtained using Rubin’s rules. The multiple-imputation analysis was prespecified as a sensitivity analysis to assess the robustness of the mortality model to missing gestational age; if the estimates differed materially from the primary complete-case analysis, the discrepancy was interpreted as uncertainty related to the missing-data assumption rather than as a replacement of the primary inference. 

Overall model fit was assessed using the omnibus likelihood ratio test and Nagelkerke’s R^2^. Model calibration was assessed using the Hosmer–Lemeshow goodness-of-fit test; discrimination was quantified by the area under the receiver operating characteristic curve (AUC), derived from predicted probabilities. The association between diagnosis and prenatal suspicion was assessed using the Fisher–Freeman–Halton exact test (Monte Carlo simulation with 10,000 samples) due to sparse cell counts [22].

Temporal trends in annual CGIM counts were examined using Poisson regression with the calendar year as a continuous predictor and the logarithm of annual paediatric surgical admissions as an offset term, thereby modelling hospital-based admission rates rather than population incidence. Because only five annual observations were available, this analysis was considered exploratory and intended to describe hospital-based temporal patterns rather than establish a robust secular trend. Goodness-of-fit statistics were examined to assess potential overdispersion. All statistical tests were two-tailed, and a *p*-value ≤ 0.05 was considered statistically significant unless otherwise specified.

### 2.8. Ethical Considerations

This study was conducted in accordance with the Declaration of Helsinki and was approved by the local institutional ethics committee (project identification code 18879, date of approval 6 June 2024) [23].

## 3. Results

### 3.1. Study Population and Hospital Burden

From January 2020 to December 2024, there were 8297 paediatric surgical admissions at our tertiary centre. ICD-10 screening identified 251 records as potentially eligible. Following a detailed manual chart review, 175 met the eligibility criteria, and 76 were excluded for various reasons. We expanded manual screening of operative notes for the same study period and identified an additional 56 eligible patients, yielding a final cohort of 231 children (Figure 1). No additional exclusions were made after eligibility was confirmed. The 231 patients accounted for 332 hospital admissions (median 1 per patient, range 1–7), representing 3.99% of all surgical admissions during the study. Most children had a single admission (n = 185, 80.1%), whereas 47 (19.9%) required two or more admissions. The proportion requiring at least two admissions differed by embryological segment (χ2 (2) = 61.89, *p* < 0.001), with hindgut malformations having the highest rate of repeated hospitalisations (57.1%), when compared with foregut (7.5%) and midgut anomalies (9.8%). The number of admissions per patient varied significantly across embryological segments (Kruskal–Wallis H(2) = 64.31, *p* < 0.001), with a higher admission burden among hindgut defects. Among patients with multiple admissions (n = 47), the follow-up period ranged from 1 to 54 months (median = 7 months, IQR 3–19 months). The follow-up period differed significantly across embryological segments (Kruskal–Wallis H(2) = 8.97, *p* = 0.011), with a longer follow-up in hindgut compared to midgut anomalies.

**Figure 2:**
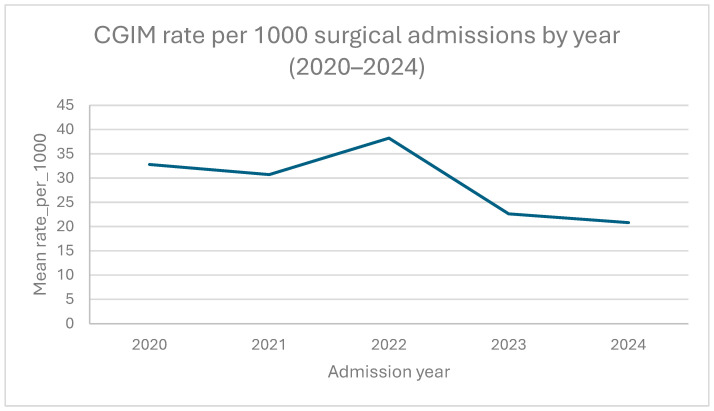
Annual CGIM admission rates per 1000 paediatric surgical admissions—index admission only, 2020–2024.

Annual CGIM case counts ranged from 40 to 56 cases/year (2020–2024). Because annual paediatric surgical admissions increased over the study period (from 1220 in 2020 to 2114 in 2024), annual CGIM admission rates ranged from 20.8 to 38.2 per 1000 surgical admissions. An exploratory Poisson model yielded a lower hospital-based admission rate over the calendar year (rate ratio per year 0.88, 95% CI 0.81–0.97; *p* = 0.008) (Figure 2). However, because this analysis was based only on five annual observations and total surgical admissions increased during the study period, this result should be interpreted as exploratory and hypothesis-generating rather than confirmatory. Goodness-of-fit statistics suggested overdispersion (Pearson χ2/df = 2.06; deviance/df = 1.91), further supporting a cautious interpretation of these results as exploratory rather than confirmatory.

Because of the retrospective design, data completeness varied across variables; all descriptive analyses used available case denominators, while multivariable analyses were restricted to complete cases, as pre-specified. Supplementary Table S1 summarises variable-level data completeness and includes only variables with incomplete data (i.e., denominator <231).

### 3.2. Baseline Demographics and Perinatal Characteristics

Of the 231 patients included, 136 (58.9%) were male, yielding a male-to-female ratio of 1.43:1. Birth weight ranged from 1.40 kg to 4.52 kg, with a median of 2.9 kg (IQR 2.52–3.28 kg). The median gestational age was 38 weeks (IQR, 36–39 weeks). Prematurity was observed in 53/190 of patients (27.9%). The median Apgar score was eight (IQR, 8–9) among those with available data (177/231). Rural residence predominated (67.5%), and patients were referred from across northeastern Romania (Supplementary Table S2). The distribution of cases by residence environment was consistent across counties; no statistically significant association was observed between county and residence environment (χ2 (8) = 3.07, *p* = 0.93), indicating a homogeneous urban–rural pattern.

Caesarean section was the most frequent mode of delivery (*n* = 95, 41.1%), followed closely by natural delivery (*n* = 88, 38.1%). Natural births occurring outside the hospital were uncommon (*n* = 6, 2.6%). Data on maternal age were available for 169 of 231 patients (73.2%). Among these, most mothers were aged 20–35 years (*n* = 127/169, 75.1%). Women aged 35 years or older accounted for 17.2% (29/169), while those younger than 20 years accounted for 7.7% (13/169).

Maternal comorbidity information was available for 180 of 231 (77.9%) mothers. Among these, any maternal comorbidity was recorded in 36/180 (20%), including chronic conditions in 21/180 (11.7%), and pregnancy-related complications in 26/180 (14.4%) (categories not mutually exclusive).

### 3.3. Diagnostic Spectrum and Embryological Distribution

**Figure 3:**
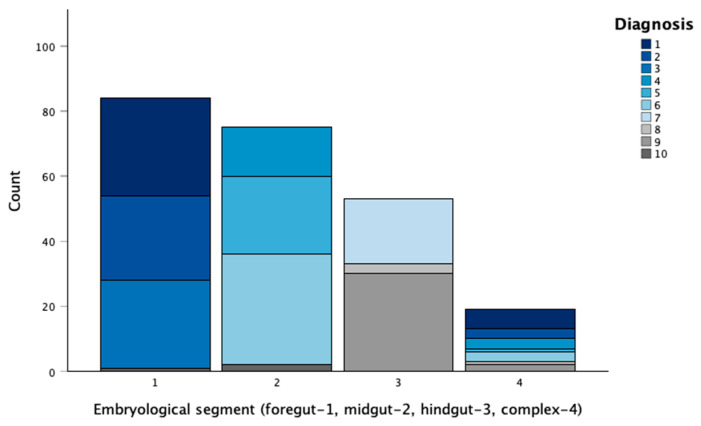
Diagnostic distribution by embryological segment, including complex cases.

A broad spectrum of congenital gastrointestinal anomalies was identified in the cohort (Figure 3). The most frequent index diagnosis was a persistent omphalomesenteric duct anomaly (*n* = 37, 16%), followed by oesophageal atresia with or without tracheoesophageal fistula (*n* = 36, 15.6%) and anorectal malformation (*n* = 32, 13.9%). Together, these categories comprised 45.5% of the cohort. Additional common diagnoses included duodenal atresia or stenosis (*n* = 29, 12.6%), hypertrophic pyloric stenosis (*n* = 27, 11.7%), and jejuno-ileal atresia or stenosis (*n* = 25, 10.8%). Less frequent conditions were Hirschsprung disease (*n* = 20, 8.7%) and intestinal malrotation with or without volvulus (*n* = 18, 7.8%). Cloacal malformations and other anomalies—such as a duplication cyst and gastric volvulus—were rare, occurring in four (1.7%) and three (1.3%) patients, respectively.

The congenital gastrointestinal malformations were unevenly distributed by embryological segments; they were most frequently located in the foregut (93/231, 40.3%), followed by the midgut (*n* = 82/231, 35.5%) and the hindgut (*n* = 56/231, 24.2%). This anatomical classification was analysed separately from phenotypic complexity and etiologic background. Complex diagnoses were established for 19/231 patients (8.2%).

### 3.4. Timing of Postnatal Diagnosis

The median age at diagnosis was 2 days (IQR 0–46; minimum–maximum 0–6463; Table 1). Because the distribution of age at diagnosis was right-skewed, particularly for persistent omphalomesenteric duct remnants, Table 1 presents age at diagnosis as the median (IQR), with minimum–maximum values. Most cases were diagnosed in the neonatal period (*n* = 161, 69.7%), including 143 cases (61.9%) detected in the early neonatal period. Diagnosis during infancy (1–12 months of age) was established in 35 patients (15.2%). The remaining 35 patients (15.2%) were diagnosed beyond one year of age. Among these, 9 cases (3.9%) occurred in early childhood (1–3 years), 3 (1.3%) in preschool-aged children (4–6 years), 11(4.8%) in school-aged children (7–12 years), and 12 (5.2%) during adolescence (13–18 years). In the subgroup of patients diagnosed under 1 year of age (*n* = 196), no significant differences in age at diagnosis were observed across embryological segments (Kruskal–Wallis H(2) = 0.12, *p* = 0.942). The Bonferroni-adjusted pairwise comparisons were likewise non-significant.

Age at diagnosis varied significantly across diagnostic groups (Kruskal–Wallis H(9) = 141.78, *p* < 0.001). The Bonferroni-adjusted post hoc Mann–Whitney U tests (approximate a = 0.001) identified a consistent early–intermediate–late gradient, with neonatal atresias and cloacal malformations presenting earlier, malrotation and anorectal malformations showing an intermediate timing of diagnosis, and omphalomesenteric duct anomalies, pyloric stenosis, and Hirschsprung disease presenting later. A similar gradient was observed when cases were stratified by embryological segment, excluding complex anomalies (Kruskal–Wallis H(2) = 23.47, *p* < 0.001). Post hoc testing showed an earlier diagnosis of foregut anomalies than midgut anomalies (U = 1850.5, *p* < 0.001) and a later diagnosis of midgut anomalies than hindgut anomalies (U = 1262.0, *p* < 0.001).

### 3.5. Prenatal Suspicion

Antenatal care information was available for 190 of 231 patients (82.3%). Among these, ultrasound reports were documented for 138 (59.7%) pregnancies, and an ultrasound was not performed in 52 (22.5%). Antenatal care information was not documented for 41/231 (17.7%) cases. Prenatal suspicion of a gastrointestinal malformation was documented in 41/231 pregnancies (17.7%). This represented 21.6% of the cases with available antenatal information (41/190) and 29.7% of the cases with documented ultrasound examinations (41/138). These proportions are hospital-based and reflect pregnancies resulting in postnatal admission to our surgical centre. Pregnancies ending in a termination of the pregnancy for foetal anomaly or foetal demise, and parentally suspected cases not transferred to our centre, are not captured. Additionally, these measures reflect documented prenatal suspicion captured in tertiary-surgical centre medical records and should not be interpreted as the true antenatal detection rate in the source population. Because antenatal documentation was unavailable for 41/231 cases, the observed documented prenatal suspicion proportion of 17.7% should be interpreted as a conservative lower estimate. In the deterministic scenario analysis, this proportion would remain 17.7% if none of the 41 undocumented cases had true prenatal suspicion, would increase to 26.4% (61/231) if 20 had true prenatal suspicion, and would reach 35.5% (82/231) if all 41 had true prenatal suspicion. 

Logistic regression analyses were restricted to patients with a complete antenatal information (*n* = 190). The primary prenatal suspicion model included four slope parameters (syndromic status, complex CGIM, and two indicator terms for embryological segment) and was fitted to complete cases (*n* = 190, prenatal suspicion = 41), yielding an events-per-parameter (EPP) of 41/4 = 10.25. In the univariate analysis, documented syndromic status was associated with increased odds of prenatal suspicion; however, the association did not reach statistical significance (OR 1.92, 95% CI 0.8–4.65; *p* = 0.146). 

In the primary multivariable model, syndromic status remained positively associated with prenatal suspicion (OR 2.19; 95% CI 0.81–5.96, *p* = 0.124). Compared with foregut anomalies, midgut defects were significantly less likely to be suspected on a prenatal ultrasound (OR 0.15, 95% CI 0.03–0.67; *p* = 0.013), whereas hindgut anomalies showed no significant association. A complex CGIM was not independently associated with prenatal suspicion (OR 1.59, 95% CI 0.43–5.94; *p* = 0.486). Model calibration was adequate (Hosmer–Lemeshow *p* = 0.837), with modest explanatory capacity (Nagelkerke R^2^ = 0.161).

In a sensitivity analysis restricted to patients with a single gastrointestinal malformation (*n* = 173/190), documented syndromic status was significantly associated with prenatal suspicion (OR 2.99, 95% CI 1.04–8.59; *p* = 0.041), with an effect size similar to that in the primary analysis. An interaction term between syndromic status and a complex CGIM was unstable (only five patients were in the combined subgroup), precluding reliable interpretation and conclusions. 

Prenatal suspicion differed significantly according to diagnosis (Fisher–Freeman–Halton exact test, Monte Carlo *p* < 0.001, Cramer’s V = 0.581). The highest proportions were observed in jejuno-ileal atresia (15/25, 60%) and duodenal atresia (14/29, 48%). Documented prenatal suspicion was also present in oesophageal atresia with or without tracheoesophageal fistula (8/36, 22.2%). No documented prenatal suspicion on routine ultrasounds was recorded in hypertrophic pyloric stenosis, malrotation, Hirschsprung disease, anorectal malformations, or other rare anomalies. Detailed diagnosis-specific rates are provided in Supplementary Table S3. 

No significant temporal trend in prenatal suspicion was observed across the calendar year (OR per year 0.91; *p* = 0.390) or between the early and later study periods (OR 1.16, 95% CI 0.53–2.55; *p* = 0.705). Among the pregnancies with documented prenatal suspicion (*n* = 41), gestational age at suspicion was available for 24/41 patients (58.5%), with a median of 28.5 weeks’ gestation (IQR 24.0–32.0).

The spectrum of non-gastrointestinal conditions documented on prenatal ultrasound (*n* = 138) was heterogeneous and included structural cardiac defects (7, 5.1%; e.g., atrioventricular canal, coarctation of the aorta, tetralogy of Fallot), urogenital anomalies (5, 3.6%; e.g., renal agenesis, hydronephrosis), neurological abnormalities (1, 0.7%; microcephaly), and chromosomal abnormalities (3, 2.2%; e.g., trisomy 21). Alterations in amniotic fluid volume were documented in 45/190 pregnancies (23.68%), most commonly polyhydramnios (*n* = 42/45, 93.33%). Additional investigations were undertaken in six pregnancies, all following ultrasound screening findings. These included amniocentesis in four cases—one combined with therapeutic amniotic fluid reduction—and foetal magnetic resonance imaging was used in three cases.

### 3.6. Phenotypic Presentation: Associated Gastrointestinal and Non-Gastrointestinal Congenital Anomalies

Beyond the ten anatomical index-diagnosis groups, the cohort showed substantial heterogeneity in phenotypic presentation. Clinically complex malformations, as defined a priori to reflect surgical severity were identified in 19/231 patients (8.2%). Within the complex subgroup, diagnostic pairings are presented descriptively in Table 2. The most frequent associations were oesophageal atresia with cloacal malformation (n = 3) or duodenal atresia (n = 2), and persistent omphalomesenteric duct remnants with abdominal wall defects (n = 3). All remaining combinations occurred only once. Malformation complexity was not associated with embryological segment (χ2(2) = 0.882, *p* = 0.643).

Associated non-gastrointestinal congenital anomalies were present in 70.1% of patients (*n* = 162/231). The number of associated anomalies per patient ranged from 0 to 5, with a median of one (IQR 0–2) (Supplementary Table S4). The burden of associated non-gastrointestinal anomalies differed across primary diagnostic categories (Kruskal–Wallis χ2 (9) = 38.14, *p* < 0.001). In post hoc pairwise comparisons with a Bonferroni adjustment, oesophageal atresia with or without tracheoesophageal fistula and duodenal atresia (both with a median of 2 [IQR 2]) were associated with a higher anomaly burden than persistent omphalomesenteric duct remnants (median 0 [IQR 1]) that were typically isolated anomalies. These findings indicate that anatomical diagnosis and phenotypic complexity are related, but not interchangeable, and support broader screening in foregut and clinically complex malformations. 

Cardiac anomalies were the most common associated non-gastrointestinal anomalies (51.1%), followed by urogenital (24.7%) and orthopaedic anomalies (17.3%); respiratory (4.8%) and endocrine/immune anomalies (3.9%) were less frequent (Table 3).

A documented etiologic or syndromic diagnosis was present in only 30 patients (13% of the cohort). Among these, 18 (7.8%) had chromosomal abnormalities (mostly trisomy 21), 1 (0.4%) had a known monogenic syndrome (achondroplasia) and 1 (0.4%) had a teratogenic syndrome (alcohol foetal syndrome). Six patients (2.6%) had recognised non-genetic malformation sequences (VACTERL association [*n* = 5] and Pierre Robin sequence [*n* = 1]). The remaining 201 (87%) patients had no identified syndrome or etiologic diagnosis. Other syndromic cases were rare and included achondroplasia (*n* = 1, 0.4%), foetal alcohol syndrome (*n* = 1, 0.4%), trisomy 18(*n* = 1, 0.4%), Pierre Robin sequence (*n* = 1, 0.4%), chromosomal 10q deletion syndrome (*n* = 1, 0.4%), and plurimalformative syndromes (*n* = 3, 1.3%).

In most patients, no specific genetic or syndromic diagnosis was documented. These cases were therefore interpreted as non-syndromic, with presumed multifactorial or unknown aetiology. However, this classification should be interpreted cautiously as genetic assessment was not performed systematically in this retrospective cohort, and these categories reflect documented diagnoses rather than the real underlying etiological diagnosis. 

### 3.7. Mortality and Outcomes

In-hospital mortality was 13% (*n* = 30/231). Of these, 17 deaths (56.7 %) occurred within the first 30 days of admission, and 13 (43.3 %) thereafter. Mortality varied significantly across embryological segments (χ2(2) = 15.83, *p* < 0.001; Cramer’s V = 0.262), with the highest mortality in foregut anomalies (23.7%, 22/93), followed by hindgut malformations (7.1%, 4/56) and midgut defects (4.9%, 4/82) with substantially lower mortality. Female patients had higher mortality rates than males (21.1% vs. 7.4%), corresponding to increased odds of death (OR = 3.36, 95% CI: 1.49–7.56). However, sex was not included in the primary multivariable model due to limited events-per-variable and was examined in the secondary analysis. In an unadjusted analysis, clinically complex anomalies were associated with markedly higher odds of death (OR 4.79, 95% CI 1.72–13.40; *p* = 0.003). 

**Figure 4:**
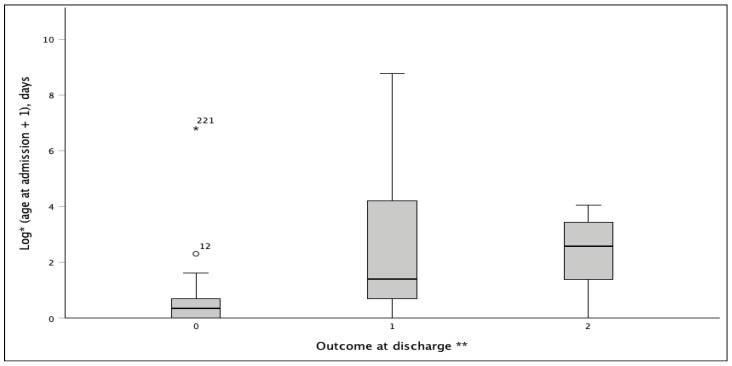
Age at index admission by discharge outcome. Footnote * Boxplots show the median and interquartile range of age at index admission; whiskers extend to 1.5 × IQR. Open circles indicate mild outliers, while asterisks indicate extreme outliers; numbers beside these symbols represent case identifiers. Because age at admission was markedly right-skewed, the y-axis is displayed on a logarithmic. ** Discharge outcome categories: 0—death, 1—discharged alive, 2—transferred to another facility.

Age at first admission differed significantly by discharge outcome (nonparametric comparison, *p* < 0.001). Patients who died were admitted at a younger age compared to those discharged alive or transferred (Figure 4). This pattern indicates that mortality was concentrated among children presenting very early in life, particularly severe neonatal cases. This observation is consistent with the higher mortality observed in foregut and clinically complex malformations and with the independent association between prematurity and in-hospital mortality. Given the marked right-skew in age, analyses were performed using nonparametric tests and visualised on a logarithmic scale. Because gestational age was missing for 41/231 patients, the primary multivariable mortality model was conducted as a complete-case analysis (*n* = 190, deaths = 27). The model included four slope parameters (prematurity, complex congenital gastrointestinal malformations [yes/no], and two indicator terms for embryological segment), corresponding to an events-per-parameter (EPP) of 27/4 = 6.75.

In the primary multivariable model (complete-case *n* = 190), prematurity was independently associated with higher odds of death (OR 2.90, 95% CI 1.14–7.42, *p* = 0.026). Model performance was acceptable (omnibus χ2(4) = 25.45, *p* < 0.001, Nagelkerke R^2^ = 0.224), with borderline calibration (Hosmer–Lemeshow *p* = 0.049) and moderate discrimination (AUC 0.708). Unadjusted associations were strong; in the complete-case multivariable model, prematurity remained independently associated with increased odds of in-hospital mortality, whereas the association with complex malformations was attenuated and did not retain statistical significance.

Multiple imputation (MI; *n* = 231) was performed as a prespecified sensitivity analysis for missing gestational age. In this secondary analysis, the adjusted estimate for complex congenital gastrointestinal malformations was larger and reached statistical significance (pooled adjusted OR 6.21, 95% CI 1.92–20.08, *p* = 0.002), while prematurity (pooled adjusted OR 2.15, 95% CI 1.21–7.88, *p* = 0.019), and midgut anomalies (pooled adjusted OR 0.15, 95% CI 0.047–0.474, *p* = 0.001) also showed significant associations with mortality. Because the primary complex-case model showed attenuation of the complex CGIM estimate with a loss of statistical significance, the MI results are interpreted as sensitivity findings only.

A secondary model including embryological segment and sex (*n* = 231) showed that midgut anomalies (adjusted OR 0.17, 95% CI 0.05–0.52, *p* = 0.002), and hindgut anomalies (adjusted OR 0.24, 95% CI 0.08–0.76, *p* = 0.015) had lower mortality than foregut defects, while female sex remained independently associated with death (adjusted OR 3.37, 95% CI 1.45–7.80, *p* = 0.005). Because sex was evaluated in a secondary analysis model rather than in the prespecified primary mortality model, this finding should be interpreted cautiously. Model calibration was good (Hosmer–Lemeshow *p* = 0.582). Adjustment for sex did not materially alter the association between embryological segment and mortality. 

Clinically complex malformations had significantly higher mortality rates compared to non-complex defects (36.8% vs. 10.8%; *p* = 0.005). Associated extra-intestinal anomalies and the study period were significantly associated with mortality in unadjusted logistic regression (both *p* < 0.001); however, these variables were not included in the multivariable model due to events-per-variable limitations. Their independent effects therefore could not be assessed reliably in this cohort.

Of the total cohort, 185 (80.08%) of the patients experienced a single hospital admission, whereas 47 (19.9%) required more than one documented admission during the study period. 

## 4. Discussion

This study describes the contemporary hospital-based overview of congenital gastrointestinal malformations managed at a tertiary referral centre in Eastern Europe between 2020 and 2024. The findings underscore the heterogeneity of these conditions across three distinct, but complementary dimensions: anatomical diagnosis, phenotypic complexity, and etiologic background. In our cohort, prenatal ultrasound suspicion remained low and dependent on the underlying diagnosis, associated extra-intestinal anomalies were frequent, and mortality was concentrated in clinically complex and foregut presentations, with prematurity remaining independently associated with death. 

Conducted in a tertiary pediatric surgical centre without obstetric services, this study reflects a referral-based cohort rather than the full regional population. Neonatal cases were included postnatally after diagnosis or suspicion, clinical stabilization, and transfer from a wide maternity network. Thus, the findings capture the structure and limitations of the regional prenatal detection and postnatal referral pathways, not population prevalence or prenatal detection. This context may explain the low prenatal suspicion rate and predominance of severe, complex cases. 

The Poisson regression model estimated a lower hospital-based CGIM admission rate over calendar years; however, this should not be interpreted as evidence of a secular decline. Given the small number of annual observations, the increase in total surgical admissions, and evidence of overdispersion, the observed pattern may reflect changes in service volume, referral pathways, or case mix rather than underlying disease occurrence. In tertiary settings, admissions can fluctuate with changing referral pathways and surgical case-mix, especially during periods of healthcare disruption. Therefore, our data do not support a definitive statement that CGIM rates declined during the study period; rather, they suggest a possible downward hospital-based pattern that requires confirmation in larger multicentre or registry-based datasets [24,25].

In our cohort, the observed prenatal suspicion rate was low overall and strongly dependent on the underlying diagnosis. Suspicion was highest for duodenal and jejuno-ileal atresia, consistent with previous reports showing better prenatal detection of obstructive anomalies associated with more recognizable sonographic signs, such as polyhydramnios and bowel dilatation [8,26,27]. By contrast, no prenatal suspicion was documented for congenital hypertrophic pyloric stenosis, Hirschsprung disease, or anorectal malformations, which is expected given that these conditions usually become clinically apparent after delivery [28,29]. A European multi-registry analysis reported higher detection rates for a duodenal obstruction compared to oesophageal atresia or a large-bowel obstruction [26]. 

European registry studies have reported higher prenatal detection for a duodenal obstruction (40–50%) than for oesophageal atresia (30–40%). In our cohort, documented prenatal suspicion was 14/29 (48.3%) for duodenal atresia/stenosis and 8/36 (22.2%) for oesophageal atresia with or without tracheoesophageal fistula. Thus, documented prenatal suspicion for oesophageal atresia in our cohort was lower than the published European estimates, but was not absent. Because our data are derived from paediatric surgical records rather than antenatal registries, these values should be interpreted as documented prenatal suspicion captured in transferred medical records, not a true population-level prenatal diagnosis. 

This discrepancy likely reflects incomplete antenatal documentation. We provide the detailed prenatal suspicion rates by diagnosis in Supplementary Table S3. Prenatal ultrasound suspicion also varies considerably across regions, with higher detection rates reported in Europe and lower rates in many resource-limited settings. Importantly, because prenatal suspicion in our study was derived from paediatric surgical notes, rather than from antenatal databases, the observed rate likely underestimates true prenatal suspicion or recognition and should be interpreted mainly as a measure of information transfer through referral pathways. Overall, our findings suggest that prenatal ultrasound detection remains both anomaly- and setting-dependent, reflecting not only the sonographic features of individual malformation, but also differences in imaging expertise, access to specialized prenatal care, and referral organization [8,25,26]. Enhancing prenatal diagnostic pathways may facilitate timely referral to tertiary surgical centres, optimize perinatal management, and support more effective parental counselling.

The phenotypic analysis highlights that anatomical diagnosis, phenotypic complexity, and etiologic background represent distinct but complementary concepts and should not be used interchangeably. In this study, anatomical diagnosis and embryological level helped explain where and how the malformation became clinically apparent, including whether the presentation was more likely to occur through antenatal ultrasound changes, neonatal obstruction, or later postnatal symptoms. Phenotypic complexity was used as a clinically defined measure to reflect the multiplicity of the gastrointestinal anomalies and surgical severity rather than underlying etiology. Etiologic background, when documented, clarified the burden and clustering of associated congenital anomalies, including extra-intestinal malformations. 

While foregut anomalies were more frequently associated with a greater burden of extra-intestinal malformations, this pattern likely reflects the combined influence of phenotypic complexity and broader developmental disturbance rather than anatomical site alone. Previous studies have likewise shown that associated malformations cluster more strongly with broader developmental and syndromic burden than with defect location alone [30,31,32]. Importantly, the clinically defined “complex” category used in the regression models should therefore be interpreted as an indicator of clinical and surgical severity, not as a proxy for underlying syndromic or etiological classification. This distinction is important, as clinical severity does not necessarily correspond to genetic or developmental etiology. 

In our cohort, only 13% of patients had a confirmed genetic or syndromic diagnosis, most often chromosomal anomalies such as trisomy 21. This proportion aligns with international observations: the World Health Organization notes that only a small share of congenital disorders can be linked to identifiable genetic abnormalities, while the causes of most remain unknown. Within this context, the majority of congenital gastrointestinal malformation cases in this study are best understood as isolated defects with complex, multifactorial origins. Syndromic presentations represented a small subset of the cohort. These findings underscore the heterogeneity and the predominantly non-syndromic nature of congenital gastrointestinal malformations.

Persistent omphalomesenteric duct anomalies, predominantly Meckel’s diverticulum, were the most frequently diagnosed congenital gastrointestinal anomalies in our cohort. This distribution differs from birth-defect registry data, where anorectal malformations and oesophageal atresia predominate [33,34]. The discrepancy likely reflects differences in ascertainment rather than a true difference in underlying occurrence since birth-based surveillance captures anomalies identified in the neonatal period, whereas tertiary surgical cohorts also capture malformations diagnosed later in childhood. Accordingly, later diagnosed congenital gastrointestinal malformations appear to contribute meaningfully to surgical workload.

The timing of presentation in our cohort reflects the interaction among developmental mechanisms, anatomical level, phenotypic complexity, etiology, and features of the referral system. Anomalies of the foregut and midgut, such as oesophageal and intestinal atresias, result from early disruptions in recanalization, rotation, or vacuolization and typically present with an acute neonatal obstruction, leading to earlier diagnosis. In contrast, conditions such as omphalomesenteric duct remnants and Hirschsprung disease may remain asymptomatic during the neonatal period, resulting in later presentation [7,35]. These anatomical patterns are further modified by phenotypic complexity and etiologic background, which influence the burden of associated anomalies and clinical severity. In addition, system-level factors, including variability in antenatal imaging, an incomplete transfer of prenatal medical records, and the exclusively outborn population, shape the likelihood of prenatal suspicion and referral pathways. Together, these factors help to explain the low overall antenatal detection rate, the higher detection rate of anomalies with characteristic ultrasound features, and the increased mortality among premature infants or those with complex or foregut anomalies. 

Associated congenital anomalies outside the gastrointestinal tract, especially cardiac and urogenital ones, were frequent in our cohort and occurred more often in complex and foregut malformations. This pattern can be explained given the shared developmental origins [9,36]. Clinically, these results emphasize a risk-stratified postnatal approach, with broader screening for foregut and complex anomalies.

Mortality in our cohort was highest among patients with foregut malformations and with complex presentations, in keeping with previous reports [37,38]. After adjustment, prematurity remained independently associated with mortality. These findings suggest that survival depends not only on the congenital malformation itself, but also on the underlying physiological vulnerability characteristic of premature neonates [37,39,40]. However, prematurity may also be a marker of greater clinical severity, which should be considered when interpreting the association. Associated non-gastrointestinal anomalies, such as cardiac and urogenital ones, were also more common in non-survivors, but their independent contribution could not be reliably assessed because of the small number of events. This discrepancy between the primary complete-case model and the multiple-imputation sensitivity analysis warrants careful interpretation. In the primary model, the adjusted association between complex CGIMs and mortality was attenuated and no longer statistically significant, whereas the imputed analysis yielded a larger and statistically significant estimate. This divergence may reflect informative missingness in gestational age data, particularly if missingness was related to clinical severity, referral pathway, or documentation quality. Alternatively, it may reflect model instability or overfitting, given the modest number of deaths relative to the number of model parameters and the small number of complex cases. Therefore, we do not conclude that complex CGIMs were independently associated with mortality based on the imputed analysis alone. Rather, the most robust adjusted finding was the association between prematurity and mortality, while the possible independent contribution of complex CGIMs should be considered hypothesis-generating and requires confirmation in larger multicentre cohorts.

An additional finding was the association between female sex and in-hospital mortality in the secondary model. This result should be interpreted cautiously. The evidence on sex-related outcome differences in neonatal and paediatric surgery is inconsistent and appears to be diagnosis-specific [41]. Whereas male disadvantage is more commonly reported in preterm neonatal populations, paediatric surgical studies have shown mixed results, with some reporting no clear sex effect and others identifying diagnosis-specific differences. In our cohort, the observed association may reflect residual confounding, a diagnosis-specific case mix, or chance, given the limited number of deaths and the exploratory nature of this analysis. Larger multicentre cohort studies are needed to determine whether sex has an independent prognostic role in congenital gastrointestinal anomalies. 

This study has several strengths. The inclusion of consecutive patients treated at a tertiary referral centre and the capture of diagnoses beyond the neonatal period provide insights into the overall spectrum of presenting congenital gastrointestinal anomalies that are not identified in birth-based surveillance. In addition, the analysis of diagnostic timing, embryological distribution, associated anomalies and discharge outcomes provides clinically relevant insights into the presentations and post-operative course of these conditions.

Several limitations should be considered when interpreting these findings. As a single-centre, retrospective, tertiary referral cohort, our data reflect institutional case mix rather than population incidence and may be affected by referral bias, limiting generalizability. Importantly, the absence of a live-birth denominator and linkage to antenatal or birth registries preclude an estimation of population incidence and true prenatal detection rates. Pregnancies ending in termination of the pregnancy for foetal anomaly or foetal demise, as well as prenatally suspected cases not transferred to our centre, were not captured; therefore, hospital-based proportions should not be interpreted as population prevalence or directly compared with registry estimates. In addition, the study relied on the completeness and accuracy of the clinical medical records. Prenatal suspicion may be under-ascertained in this study due to limitations in data sources. Antenatal information was available from paediatric surgical medical records, while external ultrasound reports were not consistently available, potentially leading to an incomplete detection of prenatal findings. Furthermore, the lack of linkage with the antenatal regional database restricted a comprehensive ascertainment of prenatal diagnoses. 

Collectively, these factors likely contributed to the under-ascertainment of true antenatal suspicion. Antenatal documentation was unavailable for 41/231 patients; therefore, the observed documented prenatal suspicion proportion of 17.7% (41/231) should be considered a conservative lower estimate for this hospital-based cohort. Under a midpoint scenario in which all 41 undocumented cases had undergone an ultrasound, and 20 had true prenatal suspicion, the cohort-level proportion would increase to 26.4% (61/231). If all 41 undocumented cases had true prenatal suspicion, the theoretical upper cohort bound would be 35.5% (82/231). These scenarios do not estimate the true prenatal detection rate in the source population, which remains unknown because pregnancies ending in foetal demise or termination and prenatally suspected cases not transferred to our centre were not captured. 

The multivariable analyses relied mainly on complete-case models, which may have reduced precision and introduced selection bias. However, the missing data mechanism differed across analyses. For mortality, multiple imputation of gestational age was used as a sensitivity analysis because gestational age was a standard clinical covariate with partial missingness. In contrast, prenatal variables were not imputed because missingness largely reflected an incomplete transfer of external antenatal documentation and, in some cases, structural non-applicability when no prenatal ultrasound had been performed. Under these conditions, imputation could have introduced misclassification rather than reduced bias. Nevertheless, incomplete prenatal documentation likely contributed to the under-ascertainment of prenatal suspicion. Finally, subgroup sizes were small, and residual confounding cannot be excluded. Notably, 24% of cases were identified only through medical and surgical note review and were not captured by ICD-10 code screening. This finding highlights the limitations of administrative coding for congenital gastrointestinal malformations and underscores the importance of multimodal case ascertainment in epidemiological studies.

Sex was evaluated in a secondary model because the limited number of deaths constrained the number of covariates that could be entered in the primary analysis. Accordingly, the association between female sex and mortality should be considered exploratory and potentially susceptible to residual confounding. 

Future progress in this field would benefit from the development of a national registry and improved linkage of antenatal, neonatal, and paediatric surgical data sources, together with structured long-term outcome assessments. Such efforts would enhance diagnostic surveillance, facilitate international benchmarking, improve referral pathways, and improve surgical outcomes.

## 5. Conclusions

In this five-year hospital-based tertiary referral-cohort review, congenital gastrointestinal malformations represented an important component of paediatric surgical workload, with most cases diagnosed during the neonatal period. Prenatal ultrasound suspicion remained low and varied according to anomaly type, while mortality was descriptively concentrated in foregut and complex presentations. In the primary complete-case multivariable model, prematurity remained independently associated with death; the independent effect of complex CGIMs remained uncertain and should not be inferred from imputed sensitivity analysis alone. These referral-cohort findings highlight the need to strengthen prenatal recognition, referral pathways, and coordinated data systems. They also support the need for a standardised national registry linked to antenatal and neonatal data, enabling surveillance, benchmarking, and targeted quality improvement. However, these findings represent a hospital-based referral cohort and should not be interpreted as estimates of population incidence or antenatal detection rates.

## Figures and Tables

**Table 1:** Distribution by age at diagnosis and anomaly burden.

Nr.	Diagnosis	Frequency (n, %)	Age at Diagnosis (Days), Median, IQR		Min-Max
1	Esophageal atresia +/− tracheo-esophageal fistula	36 (16%)	0 (0–1)		0–5
2	Duodenal atresia/stenosis	29 (13%)	1 (0–2)		0–9
3	Hypertrophic pyloric stenosis	27 (12%)	35 (28–47)		8–91
4	Intestinal malrotation	18 (8%)	6 (3–70)		0–4267
5	Jujuno-ileal atresia/stenosis	25 (11%)	0 (0–2)		0–8
6	Omphalomesenteric duct remnants	37 (16%)	2583 (313–5446)		0–6463
7	Hirschsprung disease	20 (9%)	21 (2–123)		1–2651
8	Cloacal malformation	4 (2%)	0 (0–0)		-
9	Anorectal malformation	32 (14%)	1 (0–2)		0–455
10	Others—duplication cyst/gastric volvulus	3 (1%)	390 (278–801)		165–1212
	**Total**	**231**			

Note: Age at diagnosis is reported as the median (IQR), and minimum–maximum values are provided to show the full observed spread.

**Table 2:** Clinically complex congenital gastrointestinal malformations: descriptive combinations of CGIM-CGIM and CGIM–abdominal wall defects (*n* = 19).

Index Diagnosis	Co-Occurring GI Diagnosis	*n*	% of Pairings (*n* = 19)	% Within Index Malformation (Row %)
Oesophageal atresia ± tracheoesophageal fistula	Duodenal stenosis/atresia	2	10.50	33.3
Oesophageal atresia ± tracheoesophageal fistula	Cloacal malformation	3	15.80	50.0
Oesophageal atresia ± tracheoesophageal fistula	Anorectal malformation	1	5.30	16.7
Duodenal stenosis/atresia	Intestinal malrotation	1	5.30	33.3
Duodenal stenosis/atresia	Persistent omphalomesenteric duct	1	5.30	33.3
Duodenal stenosis/atresia	Other (small bowel hypoplasia)	1	5.30	33.3
Intestinal malrotation	Abdominal wall defect (omphalocele/gastroschisis)	2	10.50	66.7
Intestinal malrotation	Hypertrophic pyloric stenosis	1	5.30	33.3
Jejuno-ileal stenosis/atresia	Abdominal wall defect (omphalocele/gastroschisis)	1	5.30	100
Persistent omphalomesenteric duct	Abdominal wall defect (omphalocele/gastroschisis)	3	15.80	100
Cloacal malformation	Intestinal malrotation	1	5.30	100
Anorectal malformation	Duodenal stenosis/atresia	1	5.30	50.0
Anorectal malformation	Persistent omphalomesenteric duct	1	5.30	50.0

Footnote: Due to sparse cells, pairings are reported descriptively.

**Table 3:** Organ system distribution of associated non-gastrointestinal congenital anomalies per patient (*n* = 231).

Associated Anomaly	*n* (%)	95% CI (Wilson), %
Cardiac	118 (51.1)	44.7–57.5
Chromosomal	22 (9.5)	6.4–14.0
Dermatologic	23 (10.0)	6.7–14.5
Endocrine/immune	9 (3.9)	2.1–7.2
Ophthalmologic/ENT/neurologic	19 (8.2)	5.3–12.5
Orthopaedic congenital	40 (17.3)	13.0–22.7
Respiratory congenital	11 (4.8)	2.7–8.3
Sequences/syndromes	30 (13.0)	9.3–17.9
Urogenital	57 (24.7)	19.6–30.6
Any associated anomalies (>=1)	162 (70.1)	63.9–75.7

Footnote: Data are *n* (%). Ninety-five percent confidence intervals (CIs) for proportions were calculated using the Wilson score method (denominator *n* = 231; no missing data). Categories are not mutually exclusive.

## Data Availability

The original contributions presented in this study are included in the article. Further inquiries can be directed to the corresponding author.

## Supplementary Materials

The following supporting information can be downloaded at: https://www.mdpi.com/article/10.3390/diagnostics16091408/s1. Supplementary Table S1—Completeness of study variables; Supplementary Table S2—County-Level Breakdown; Supplementary Table S3—Detailed prenatal suspicion rates by diagnosis; Supplementary Table S4—Number of associated non-gastrointestinal anomalies per patient.

## Abbreviations

The following abbreviations are used in this manuscript:

ARM	Anorectal malformation
χ2	Chi-square
CGIMs	Congenital gastrointestinal malformations
CI	Confidence interval
DA	Duodenal atresia
e^2^	Epsilon squared
EA +/− TEF	Oesophageal atresia with/without tracheoesophageal fistula
EPP	Events-per-parameter
EUROCAT	European Surveillance of Congenital Anomalies
HD	Hirschsprung disease
HPS	Hypertrophic pyloric stenosis
ICD–10	International Classification of Diseases, 10th Revision
IQR	Interquartile range
IRR	Incidence rate ratio
MI	Multiple imputation
OMD	Omphalomesenteric duct
OR	Odds ratio
SPSS	Statistical product and service solutions
VACTERL	Vertebral, anal, cardiac, tracheo-oesophageal, renal, and limb anomalies
