# Supplementary material for: Congenital Gastrointestinal Malformations in a Romanian Tertiary Centre (2020–2024): A Retrospective Cohort Study of Diagnosis, Distribution, and Outcomes
Source: Diagnostics (Basel). 2026 May 6;16(9):1408. doi: 10.3390/diagnostics16091408 (PMC13163343; doi:10.3390/diagnostics16091408)
Supplement: Supplementary file 1 [file diagnostics-16-01408-s001.zip › diagnostics-4237196-supplementary.pdf]

Supplementary Table S1 -S4

Supplementary Table S1 – Completeness of study variables

Only variables with at least one missing observation are recorded in this table. Percentages are reported relative to the total cohort (n=231) and, where applicable, relative to the corresponding subset.

VARIABLE	DOMAIN	USED IN RESULTS SECTION	APPLICABLE DENOMINATOR	RECORDS WITH DATA AVAILABLE, N	COMPLETENESS % OF TOTAL COHORT	COMPLETENESS % OF APPLICABLE SUBSET	N MISSING	MAIN REASON FOR MISSING DATA
Antenatal care	Antenatal	Antenatal data	231	190	82.3	82.3	41	Antenatal care information not documented
Antenatal suspicion of CGIM	Antenatal	Antenatal data	231	190	82.3	100	41	Antenatal diagnosis information not documented
Antenatal ultrasound	Antenatal	Antenatal data	190	138	59.7	72.6	93	Antenatal ultrasound information not documented
Apgar score	Perinatal	Baseline demographics	231	177	76.6	76.6	54	Not available in referral documents
Birth weight	Perinatal	Baseline demographics	231	185	80.1	80.1	46	Birth weight not recorded
Gestational age at antenatal diagnosis	Antenatal	Antenatal data	231	24	10,4	10,4	207	Timing of antenatal diagnosis not recorded
Maternal comorbidities	Maternal	Maternal characteristics	231	180	77.9	77.9	51	Maternal history incompletely recorded
Mode of delivery	Perinatal	Baseline demographics	231	189	81.8	81.8	42	Mode of delivery not recorded
Prematurity	Perinatal	Baseline demographics	190	190	82.3	100	41	Gestational age not recorded

Supplementary Table S2 – Distribution of patients across referral counties (table and map)

COUNTY	FREQUENCY	PERCENT %
BACAU	22	9,5
BRAILA	3	1,3
BOTOSANI	22	9,5
GALATI	5	2,2
IASI	70	30,3
NEAMT	31	13,4
SUCEAVA	37	16
VRANCEA	14	6,1
VASLUI	27	11,7
**TOTAL**	**231**	**100**

Supplementary Table S3. Documented prenatal suspicion by index diagnosis in children with congenital gastrointestinal malformations (n-231).

	Diagnosis	Documented antenatal suspicion(n)	Documented antenatal suspicion(%)	Total	Detection
1	Esophageal atresia +/- tracheoesophageal fistula	8	22,2	36	Low
2	Duodenal atresia/stenosis	14	48,3	29	Moderate-high
3	Pyloric stenosis	0	0	27	No detection
4	Intestinal malrotation	0	0	18	No detection
5	Jejunoileal atresia/stenosis	15	60	25	High
6	Omphalomesenteric duct remnants	2	5,4	37	Very low
7	Hirschsprung disease	0	0	20	No detection
8	Cloacal malformation	2	50	4	High, but very small n
9	Anorectal malformation	0	0	32	No detection
10	Others	0	0	3	No detection

Footnote: Prenatal suspicion was defined as antenatal ultrasound suspicion documented in the patient's surgical record and confirmed postnatally. This variable is different from the timing of postnatal diagnosis. For oesophageal atresia with or without tracheoesophageal fistula, documented prenatal suspicion was present in 8/36 cases (22.22%). If "diagnosis at birth" is reported elsewhere in the supplementary material, it refers to postnatal diagnosis during the birth admission and should not be interpreted as a prenatal diagnosis.

Supplementary Table S4 – Distribution of the number of associated congenital anomalies (n = 231)

Nr of associated anomalies	n (%)	95% CI (Wilson), %
0	70 (30.3)	24.7–36.5
1	78 (33.8)	28.0–40.1
2	41 (17.7)	13.4–23.2
3	31 (13.4)	9.6–18.4
4	9 (3.9)	2.1–7.2
5	2 (0.9)	0.2–3.1

Note. Data are n (%). 95% confidence intervals were calculated using the Wilson score method (denominator N = 231; no missing data).
